# Letter to the editor: recurrent symptoms of gastrointestinal tract caused by isolated endometriosis in a middle-aged female

**DOI:** 10.1007/s00384-016-2522-9

**Published:** 2016-02-03

**Authors:** Chuan Zhang, Ye Sun, Dongsheng Zhang, Yueming Sun

**Affiliations:** Department of Colorectal Surgery, The First Affiliated Hospital of Nanjing Medical University, 300 Guangzhou Road, Nanjing, Jiangsu 210029 People’s Republic of China

Dear Editor:

Endometriosis is defined as the presence of endometrial-like tissue outside the uterine cavity. Of all the cases of endometriosis, intestinal endometriosis accounting for about 10 %, the rectum and sigmoid colon are the most vulnerable position of the intestinal tract.

For doctors, the diagnosis of colorectal endometriosis is difficult, especially the differential diagnosis between this disease and other diseases such as malignancies, irritable bowel syndrome, inflammatory bowel disease, ileal Crohn’s disease, and so on, due to its unspecific symptoms. Today, a large number of these cases are found accidentally at surgery and confirmed by pathology. Here, we describe a case which has not a definite preoperative diagnosis, but the histopathological examination of the resected specimen showed ileocecal endometriosis infiltrating the external muscular layer and two of the nine lymph nodes.

## Clinical information

A 47-year-old woman was referred to our hospital because of dyschezia, abdominal pain, abdominal distension, and mild nausea, associated with constipation. During 5 years before admission, the patient had episodes of abdominal pain especially at the lower abdominal and around the navel without obvious inducement. Abdominal pain can be relieved after defecation. Passing feces is tough for her and it occurs once each 2 to 3 days. The feces, shape is thinner than normal, with mucus on the surface, sometimes with few blood.

A colonoscopy performed in the patient^’^s hometown hospital 4 months before admission, at about 11–16 cm from the anal margin, revealed a stenosis with intact and a little thickened mucosa. Histopathological examination of the sigmoid from the colonoscopy showed chronic inflammation of mucosa.

Two months earlier, the symptoms of dyschezia, abdominal pain, and abdominal distension became worse and much more frequent. However, the patient experienced no obvious improvement after antispasmodic and analgesic drugs were administered in her hometown hospital. The patient got married at the age of 26 years and had one normal labor at the age of 29 years, regular menses and no history of dyspareunia. Her last menstrual period was 1 week before and it was unremarkable. She had no past history of hypertension, diabetes, coronary heart disease, tuberculosis, or other infectious diseases. Besides, she had no operation history, radiation exposure history, or a poisonous chemical contact history. She also denied smoking and has occasional alcohol use. Her father and mother were healthy without reporting previous history of such disease. Her brother and her son were also healthy.

The abdomen looked flat with no intestinal type or peristalsis wave. Palpation revealed mild lower abdominal tenderness and no abdominal masses or enlarged lymph nodes. The bowel sounds were slightly increased. Rectal examination showed no blood or distinct mass. On the gynecological examination, her vulva, vagina, and cervix appeared to be normal. Her uterus had normal size and was anteverted.

Routine stool test was unnormal with occult blood active. Routine blood test, coagulation parameters, contagious parameters, routine urine test, liver function tests, renal function tests, AFP, CEA, CA199, and CA724 were all within normal range, whereas CA125 was twice as much the normal level.

An ultrasound colonoscopy underwent in our hospital after admission showed an impassable stenosis, covered with rough, proliferous, anabrotic, and a little hematose mucous membrane, 15 cm from the anus, and the scope could not pass through the lesion. The ultrasound scan revealed obviously thickened intestinal wall, low echo in wide range, and mucosa with uncleared level. The pathobiology again confirmed mucosal chronic inflammation with lymphoid hyperplasia.

A contrast-enhanced abdominal computed tomography scan revealed a swelling of the sigmoid wall and stenosis, and the uterine density was showed being not uniform. Concerning the duration, symptoms of incomplete intestinal obstruction, colonoscopy, and pathology reports, we thought the possibility of inflammatory bowel disease was large and could not completely rule out the possibility of tumor. In the last 2 weeks, symptoms of dyschezia became more serious; defecation was more difficult for her—it occurred once each 3–5 days, and abdominal pain, abdominal distension, vomit, nausea were worse than before, accompanied by hemafecia.

A laparoscopy performed 3 days later revealed obviously a sigmoid mass near the peritoneal fold, 4 × 3 cm in size; adhesion between the neck of the uterus and rectum; and adhesion between the left ovary, fallopian tube, and the surroundings. No other organs were invaded. We resected the sigmoid at 12 cm above the tumor and 4 cm below the mass accompanied by lymph nodes in groups 241, 242, and 251 by the standard of carcinoma of the colon. Histopathological examination showed ileocecal endometriosis affecting the external muscular layer and two of the nine lymph nodes. The postoperative course was uneventful; the patient recovered well and left the hospital 8 days later. She was on regular follow-up and suffered no recurrence of the same symptoms one and half years later.

## Discussion

Endometriosis is characterized as pelvic or extrapelvic, and the gastrointestinal tract is the most common site of the extrapelvic implantation sites. Compared to ileum, appendix, transverse colon, and cecum endometriosis usually arise in the rectosigmoid in 80 % of these cases.

The etiology of endometriosis is still elusive and among the theories explaining the pathogenesis of endometriosis, the most widely accepted theory is Sampson’s “retrograde menstruation” theory: endometrial tissue regresses through the fallopian tubes, then implants and grows on the serosal surface of extra-uterine organs.

Gastrointestinal tract endometriosis usually takes the form of asymptomatic small serosal implants. Influenced by hormone during menstruation, these implants may proliferate and bleed cyclically, bringing about non-specific symptoms like vomiting, diarrhea, constipation, pelvic pain, pencil-like stool, cyclical hematochezia, frequent urination, pain during defecation, dyspareunia, infertility, and abdominal mass. Forty percent of the patients present symptoms in a cyclic manner, which are usually related with menses. In our patient, symptoms relapsed irregularly and were not related with menses. Endometriosis infiltrating the muscularis propria and after cyclic proliferation, sloughing, and bleeding, inflammation and fibrosis appear and grow into the lumen, leading to obstruction. Invasive bowel endometriosis can present as bowel obstruction in an acute, chronic, or intermittent manner. The true incidence of endometriosis causing bowel obstruction is unknown, although complete obstruction of the bowel lumen occurs in less than 1 % of cases.

Bowel symptoms can present a confusing clinical picture and masquerade a wide spectrum of disease processes, including irritable bowel syndrome, infectious disease, ischemic colitis, inflammatory bowel disease, ileocolonic intussusception, appendicitis, and malignancy.

Physical examination cannot often provide sufficient clues for the diagnosis; hence, a timely and accurate preoperative diagnosis is often delayed. Imaging studies, such as abdominal CT, contrast-enhanced abdominal CT, magnetic resonance imaging (MRI), endoscopic ultrasound, and transvaginal sonography, are widely applied for detecting alterations in the intestinal wall. Among these ones, MRI appears to be the most sensitive technique for colorectal endometriosis with a positive predictive value of approximately 77.5–92.6 %. CT is not the primary imaging modality for evaluation of bowel endometriosis. Transvaginal ultrasound has a reported sensitivity of 91 % and specificity of 98 %. Endoscopy usually provide no valuable results for a definitive pathologic diagnosis because of the intact mucosa due to the reason that endometriosis involves the deep layers of the bowel wall in general. But in our practical work, we still use endoscopy routinely for differential diagnosis, whether it is endometriosis or malignant lesions or other diseases.

Additionally, most cases are found accidentally at surgery and the gold standard for the diagnosis is laparoscopy or laparotomy, by which we can evaluate both the genital and intestinal tracts more completely and accurately.

Treatment options comprise hormonal and surgical options. But we should choose the appropriate method according to patient’s age, fertility plan, the stage of the disease, and complications of the disease. Hormonal therapy can be considered when the disease has no symptoms of obstruction and we can apply hormones like danazol, gonadotrophin-releasing hormone (GnRH) analogs, and high-dose progestins. But these medicines are not recommended for patients who desire to become pregnant.

Surgery is the choice of treatment for intestinal endometriosis when there are symptoms as intestinal obstruction, bleeding, severe pain, and if cancer cannot be excluded. Nowadays, we use laparoscopy to resect the intestinal lesions completely to prevent the tumor growth more often than laparotomy unless there are complicated adhesions.

